# Synergistic Action of D-Glucose and Acetosyringone on *Agrobacterium* Strains for Efficient *Dunaliella* Transformation

**DOI:** 10.1371/journal.pone.0158322

**Published:** 2016-06-28

**Authors:** Ramachandran Srinivasan, Kodiveri Muthukalianan Gothandam

**Affiliations:** School of Bio-Sciences and Technology, VIT University, Vellore – 632 014, Tamil Nadu, India; Instituto de Biología Molecular y Celular de Plantas (IBMCP), SPAIN

## Abstract

An effective transformation protocol for *Dunaliella*, a β-carotene producer, was developed using the synergistic mechanism of D-glucose and Acetosyringone on three different *Agrobacterium* strains (EHA105, GV3101 and LBA4404). In the present study, we investigated the pre-induction of *Agrobacterium* strains harboring pMDC45 binary vector in TAP media at varying concentrations of D-glucose (5 mM, 10 mM, and 15mM) and 100 μM of Acetosyringone for co-cultivation. Induction of *Agrobacterium* strains with 10 mM D-glucose and 100 μM Acetosyringone showed higher rates of efficiency compared to other treatments. The presence of GFP and HPT transgenes as a measure of transformation efficiency from the transgenic lines were determined using fluorescent microscopy, PCR, and southern blot analyzes. Highest transformation rate was obtained with the *Agrobacterium* strain LBA4404 (181 ± 3.78 cfu per 10^6^ cells) followed by GV3101 (128 ± 5.29 cfu per 10^6^ cells) and EHA105 (61 ± 5.03 cfu per 10^6^ cells). However, the *Agrobacterium* strain GV3101 exhibited more efficient single copy transgene (HPT) transfer into the genome of *D*. *salina* than LBA4404. Therefore, future studies dealing with genetic modifications in *D*. *salina* can utilize GV3101 as an optimal *Agrobacterium* strain for gene transfer.

## Introduction

*Dunaliella salina* is a well-established natural producer of β-carotene in which synthesis of the pigment is enhanced under extreme environmental conditions, such as illumination intensity, temperature fluctuations and nutrition (depletion or limitation). Although research has yielded several expression systems for exploitation of the algae for its products, hitherto available systems possess several disadvantages in the production of recombinant products, nutraceutical by-products, biodiesel or other value added products. Regardless of the existence of several techniques for gene transformation in *D*. *salina*, such as electroporation [1], glass bead [2], biolistic gun [3], silicon carbide whiskers [4], protoplast transformation [5], microinjection [6] and PEG-mediated transformation [7], *Agrobacterium*-mediated transformation remains one of the most promising method for transient expression and stable integration of foreign gene into the algal host. Extensive work has been carried out in the development of efficient *Agrobacterium-* mediated transformation protocols for microalgae with various types of inducers[8, 9]. Unfortunately, the efficiency of the transformation is found to be low due to two potential reasons: poor induction of *Vir* genes for T-DNA transfer and species specificity of the *Agrobacterium* strains. Inducers are compounds which play a crucial role in the activation of the *Vir* gene which prompts genome-integration resulting in an overall increase in the rate of transformation efficiency [10]. Few investigations have reported *Agrobacterium-*mediated transformation in microalgae in the presence or absence of phenolic compounds acting as inducers [11, 12] and some studies have also investigated pre-induction of *Agrobacterium* with Acetosyringone (AS) and plant-derived compounds for efficient transformation in microalgae under low pH [9, 13, 14, 8].

*Agrobacterium* detects and responds to plant-derived sugars through a distinct signaling pathway involving VirA and a chromosomally encoded periplasmic protein (ChvE). ChvE mediates a sugar-induced increase in Virulence (*Vir*) gene expression through activities of the VirA/VirG two-component regulatory system [15–17]. This forms a part of an operon encoding an ABC-type transport system which is transcriptionally induced by sugar molecules [18]. ChvE mediates *Agrobacterium* chemotaxis in response to aldose monosaccharides such as galactose, glucose, arabinose, fucose, xylose, and other sugar acids which interact with VirA [19]. Expression of ChvE is regulated by the transcriptional regulator glucose/galactose-binding protein regulator (GbpR) in the presence of sugars [20]. Monosaccharide molecules involved in the activation of the *Vir* genes have resulted in the induction of ChvE to a maximum of eightfold during their absence; GbpR represses its expression [17]. From the understanding of these reports, we developed an efficient transformation protocol for *Dunaliella* by pre-induction of *Agrobacterium* strains (EHA105, GV3101and LBA4404) with D-glucose in the presence of AS.

## Materials and Methods

### Algal strain and culture conditions

Algal strain *D*. *salina* V-101 was acquired from the Centre for Advance Science Botany, Madras University, Chennai. Axenic cultures were maintained in De Walne’s medium [21]. Initially, Algal cells were grown in TAP medium containing 2.0 M concentration NaCl. Later, *Dunaliella* culture was transferred every second day into fresh TAP medium, with gradual reduction of 0.1 M from preceding concentration of NaCl every time until cells were acclimated to 0.15 M NaCl. Then, *Dunaliella* cells were maintained in both liquid and solid TAP medium containing 0.15 M for the remaining experimental studies. *Dunaliella* cells were incubated at 24 ± 1°C in a growth chamber with an illumination of 22 μmol m^−2^ s^−1^ under a 16:8-h photoperiod.

### *Agrobacterium* strains and vector transformation

**Fig 1:**
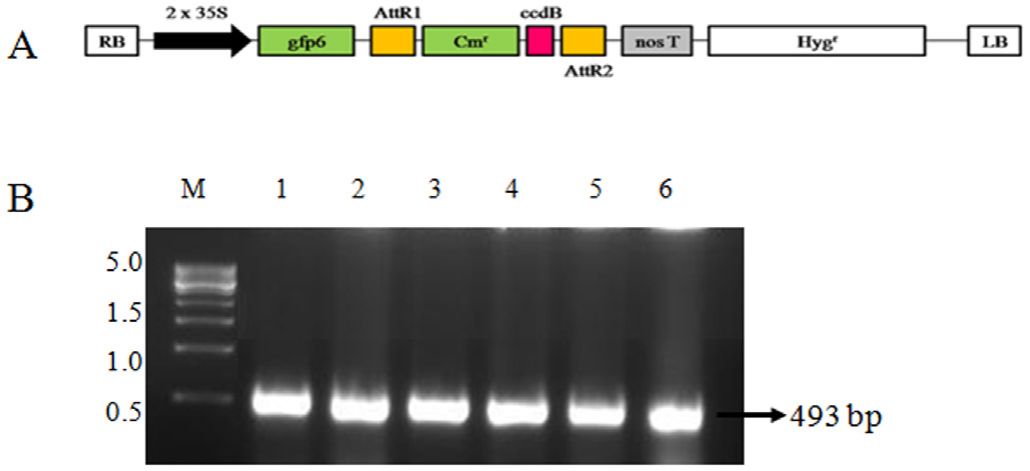
Agrobacterium strains (EHA105, GV3101 and LBA4404) harboring binary vector pMDC45. (A) Schematic map of binary vector pMDC45. RB, Right border; LB, Left border; GFP, Green fluorescent protein gene. (B) Confirmation of *Agrobacterium* strains EHA105 (Lane 1 and 2), GV3101 (Lane 3 and 4) and LBA4404 (Lane 5 and 6) with pMDC45 by colony PCR, M—500 bp Marker in kb. The gel shows the corresponding 493bp fragments obtained with GFP-specific primers.

Three strains of *A*. *tumefaciens* (EHA105, GV3101 and LBA4404) were obtained from the Indian Council of Agricultural Research (ICAR), Delhi and transformed using the binary vector pMDC45 (The Arabidopsis Information Resources). The characteristics of these *Agrobacterium* strains are explained in Supporting information, S1 Table. The vector pMDC45 harbors GFP as a reporter gene and, kanamycin and hygromycin resistant genes as selective markers which are driven by the 2xCaMV 35S promoter (Fig 1A). Selected single colonies were analyzed by colony PCR using a GFP-specific primer (S2 Table). The conditions for PCR were as follows: initial denaturation at 95°C for 5 min, 35 cycles of 94°C for 30 s, 60°C for 30 s and 72°C for 1 min, a final extension at 72°C for 10 min and hold at 4°C. PCR-positive colonies were inoculated into 5 ml of YEP medium containing 50 mg/L kanamycin and 25 mg/L rifampicin and incubated at 28°C for 48 h with shaking at 200 rpm. *Agrobacterium* strains were maintained as a glycerol stock in -80°C for further use.

### Experimental study

Co-cultivation of *D*. *salina* and pre-induced *Agrobacterium* strains were divided into six different treatments: Group I, designated as control (without AS and D-glucose); Group II, *Agrobacterium*-induced with 100 μM AS alone; Group III, *Agrobacterium*-induced with 10 mM D-glucose alone; Group IV, V and VI, *Agrobacterium*-induced with 100 μM AS with D-glucose at a concentration of 5 mM, 10 mM and 15 mM respectively.

### Induction of *Agrobacterium* strains

Single colonies of the three *Agrobacterium* strains were inoculated in LB media supplemented with 50 mg/L kanamycin and 25 mg/L rifampicin and incubated overnight at 28°C (OD 600 = 0.8 to 1). Following this, the *Agrobacterium* cultures were centrifuged at 4000 rpm for 5 min and the pellets obtained were re-suspended in induction TAP medium containing only 100 μM AS (Group II), only 10mM D-glucose (Group III) and 100 μM AS along with different concentration of D-glucose (5, 10 and 15 mM) for *Vir* gene induction at pH-5.2 (Groups IV, V and VI) [9]. The cultures were incubated at 25°C for 24 h and used for co-cultivation.

### Co-cultivation of *D*. *salina* and *Agrobacterium* strains

Co-cultivation of *Agrobacterium* with *D*. *salina* and antibiotic sensitivity test for *D*. *salina* with different antibiotics were carried out as per the protocol [11]. Twenty milliliters of log phase cultures of *D*. *salina* (0.8 to 1.0 OD at 520 nm) were spread onto TAP medium and incubated under 16 h light irradiance for a week until algal lawn formations were observed. Two hundred microliters of pre-induced *Agrobacterium* strains harboring pMDC45 in TAP medium was plated over the lawn of *D*. *salina* cells and incubated under same conditions for 48 h. Following co-cultivation, cells were scraped and re-suspended in liquid TAP medium after which they were centrifuged at 3500 rpm for 2 min. Cells were then washed thrice with TAP medium containing 500 mg/L cefotaxime with 3 mg/L hygromycin and further centrifuged at 4000 rpm for 5 min. The cell pellets obtained were suspended in TAP liquid medium and spread onto a solid TAP medium containing 3 mg/L of hygromycin. After 4–7 weeks, individual colonies were picked and spotted onto a separate plate containing 3mg/L of the selective antibiotic, hygromycin.

### Expression of GFP reporter gene in transgenic lines and wild-type

Selected transgenic lines and wild-type cells were treated with methanol:tetrahydrofuran (1:1) for 20 min to partially remove the chlorophyll content [11] and cells were washed thrice with distilled water. The cells were then examined under a Weswox optic LED fluorescent microscope FM-3000 at an excitation B filter wavelength ranges between 460 and 490 nm.

### Analysis of putative transgene in *Dunaliella* cells by PCR

Ten milliliters of selected transgenic lines and wild-type cells (0.8 to 1.0 OD at 520 nm) were harvested by centrifugation at 3500 rpm and genomic DNA was isolated using the CTAB (Cetyltrimethylammonium bromide) method [22]. Algal cells were dissolved in 500 μl of extraction buffer containing 1 M Tris-Cl (pH 8.0), 0.5 M EDTA (pH 8.0), 5 M NaCl, 2% CTAB and 0.2% β-mercaptoethanol and incubated at 60°C for 20 min. After incubation, samples were centrifuged at 10,000 rpm at 4°C. Then, DNA was extracted from the pellet using ice-cold isopropanol and dissolved in TE buffer containing 10 mM Tris-Cl (pH 7.5), 1 mM EDTA. PCR was carried out using GFP and HPT specific primers to detect the presence of specific genes in transgenic lines and wild-type cells (S2 Table). PCR conditions were as follows: initial denaturation at 95°C for 5 min, 35 cycles of 94°C for 30 s, 60°C for 30 s and 72°C for 1 min, a final extension at 72°C for 10 min and termination at 4°C. The PCR products were evaluated bygel electrophoresis on a 1% agarose gel.

### Southern analysis of transgenic lines and wild-type

Southern blot analysis was carried out to further confirm the copy number of gene integrated to the genome in transgenic lines using DIG-DNA Labeling and Detection Kit (Roche, USA) and by following the manufacturer’s instructions. Genomic DNA was isolated from transformed and wild-type cells by CTAB method [22]. Samples containing 5 μg of genomic DNA and control plasmid pMDC45 were digested with *KpnI*. The 600 bp long fragment of HPT was used to prepare a DIG-labelled probe.

### Identification of bacterial contamination in the transformed cells

Identification of *Agrobacterium* contamination in the genomic DNA of the transformed cells was analyzed using PCR for kanamycin resistant gene, (S2 Table) which lies outside of right and left border of pMDC45. PCR conditions included initial denaturation at 95°C for 5 min, 35 cycles of 94°C for 30 s, 60°C for 30 s and 72°C for 1 min, a final extension at 72°C for 10 min and hold at 4°C. pMDC45 binary vector was used as a positive control for the study.

### Long-term stability analysis of transgenic microalgae

To study the stability of transgenes, transgenic *Dunaliella* cells were sequentially subcultured for more than six months in TAP medium in the absence of hygromycin. To select stable transgenes, the transgenic algal cells were exposed to different concentration of hygromycin. The survival rate was evaluated by calculating the optical density of hygromycin tolerant *Dunaliella* cells. To confirm the transgene, DNA was isolated and analyzed for the presence of hygromycin resistant gene in the genome of algal transformants by PCR using HPT gene-specific primers (S2 Table).

### Statistical analysis

Three replicates per group were used in the experimental study and the values have been expressed in Mean ± SD. The analysis of variance (Two-way ANOVA) between *Agrobacterium* strains and each group (p<0.05) was conducted using GraphPad Prism (Version 5.0).

## Results

Binary vector pMDC45 containing HPT resistant marker and GFP as reporter gene driven by CaMV35S promoter was used in this study (Fig 1). The binary vector was successfully transformed into *Agrobacterium* strains EHA105 (Lane 2 and 3), GV3101 (Lane 4 and 5) and LBA4404 (Lane 6 and 7) using the freeze-thaw method. Selected transformed cells were screened for the presence of pMDC45 in *Agrobacterium* strains using colony PCR, with GFP-specific primer (Fig 1B).

### Effect of hygromycin and cefotaxime on the growth of *Dunaliella salina*

**Fig 2:**
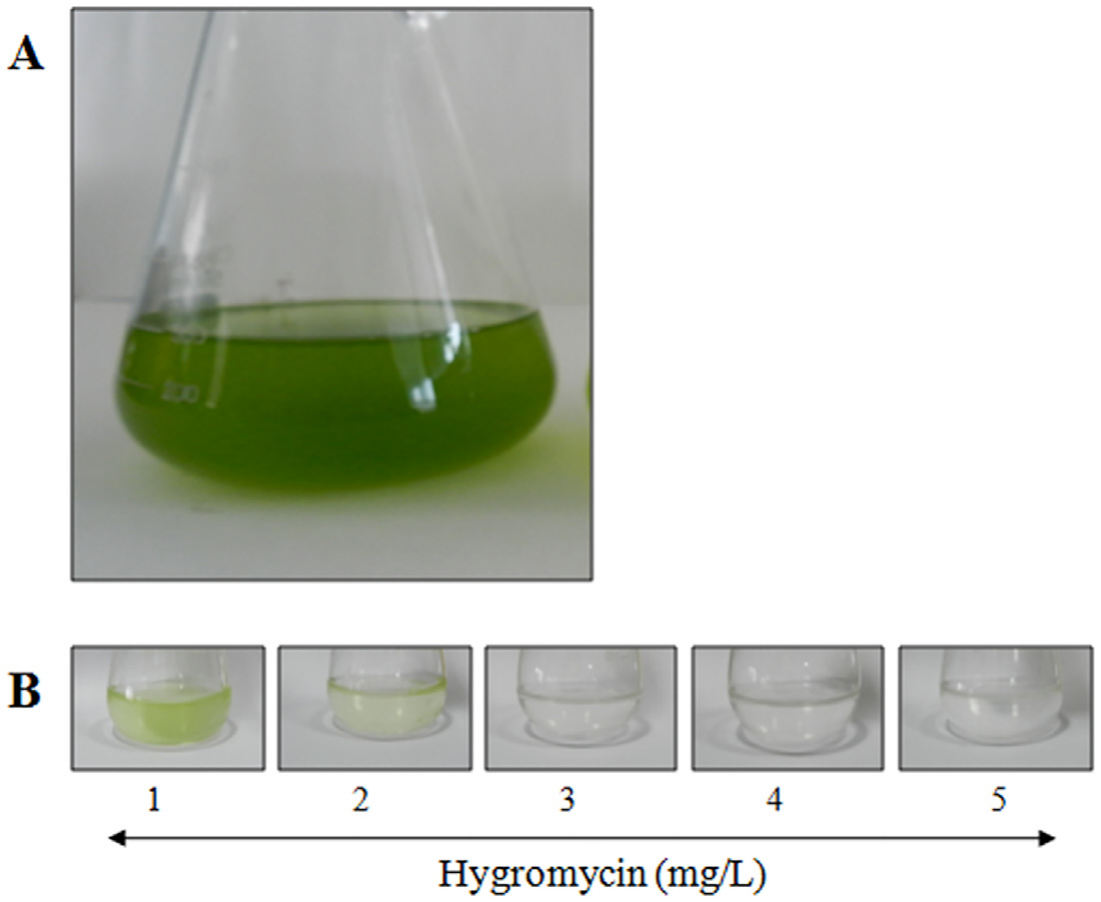
Antibiotic sensitivity and growth characteristics of *D*.*salina* in TAP medium. (A) The growth of *D*. *salina* in TAP medium containing 0.15 M NaCl by gradual reduction of NaCl concentration. (B) Antibiotic sensitivity of *D*. *salina* at different concentrations of Hygromycin (1–5 mg/L) in the TAP medium.

The growth of the algae in TAP medium containing 0.1 M NaCl with different concentrations of hygromycin is represented in the Fig 2A and 2B and the result indicates a complete growth inhibition at 3 mg/L hygromycin (Table 1). This concentration of hygromycin was used for the selection of transgenic lines, and it was observed that cefotaxime did not affect the growth of algae even at a concentration as high as 2000 mg/L in the TAP medium. Thus, 500 mg/L of cefotaxime is enough to eliminate the *Agrobacterium* strains from the transgenic lines (S3 Table).

**Table 1:** Effects of hygromycin on the growth of *D*. *salina* for the selection of transformed cells (hygromycin resistant strains).

S.No	Hygromycin concentration (mg/L)_	% Survival
1	0	100.00
2	1	59.71
3	2	12.71
4	3	0.00
5	4	0.00
6	5	0.00

Complete inhibition of cell growth was observed at a hygromycin concentration of 3 mg/L.

### Co-cultivation of *D*. *salina* and pre-induced *Agrobacterium* strains

**Fig 3:**
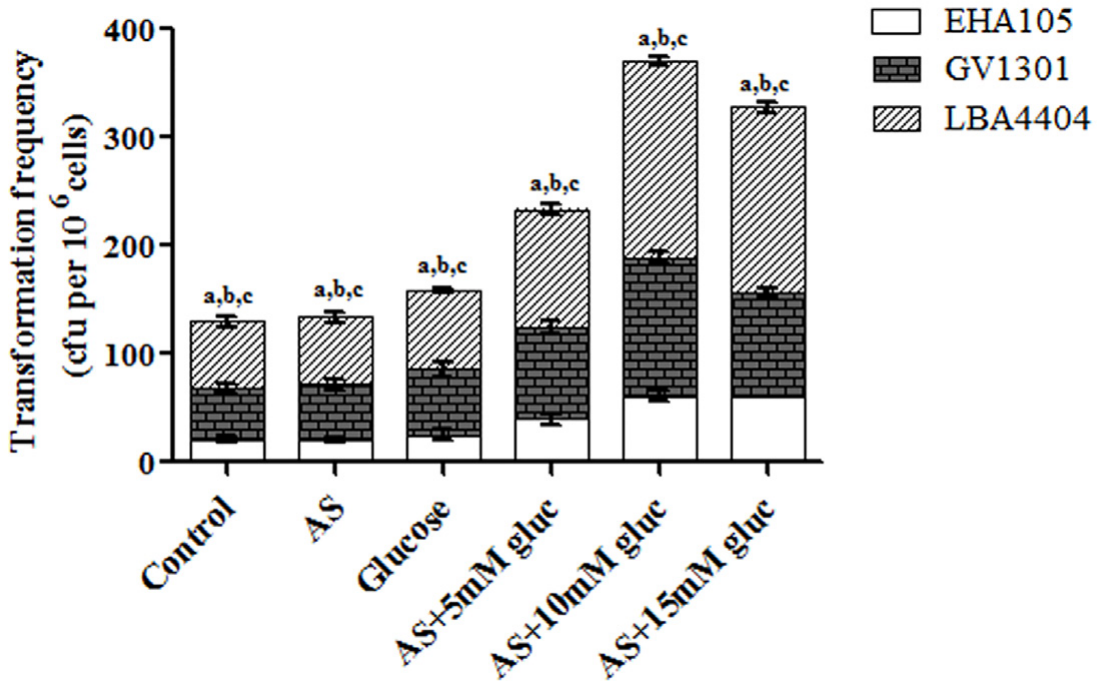
Transformation efficiency of *Agrobacterium* strains on *D*. *salina* in each group. Values are expressed as Mean ± SD (n = 3). Letters a-c represent significant values between *Agrobacterium* strains (p<0.05).

Induction of *Agrobacterium* strains was performed with different concentrations of D-glucose (5 mM, 10 mM, and 15 mM) in the presence of 100 μM AS. Further, 10 mM D-glucose alone and 100 μM AS alone were also used to infect *Dunaliella* cells by co-cultivation. *Agrobacterium* strains showed maximum algal transformation efficiency at 10 mM D-glucose concentration combined with 100 μM AS (S1 Fig). From the three strains, LBA4404 presented a significantly higher number of transformants (181 ± 3.78 cfu per 106 cells) followed by GV3101 and EHA105 (EHA105>GV3101>LBA4404). The inducer AS and glucose alone do not cause any difference in transformation efficiency compared to control (without AS) during co-cultivation (Fig 3).

### Expression of GFP reporter gene in *Dunaliella* cells

**Fig 4:**
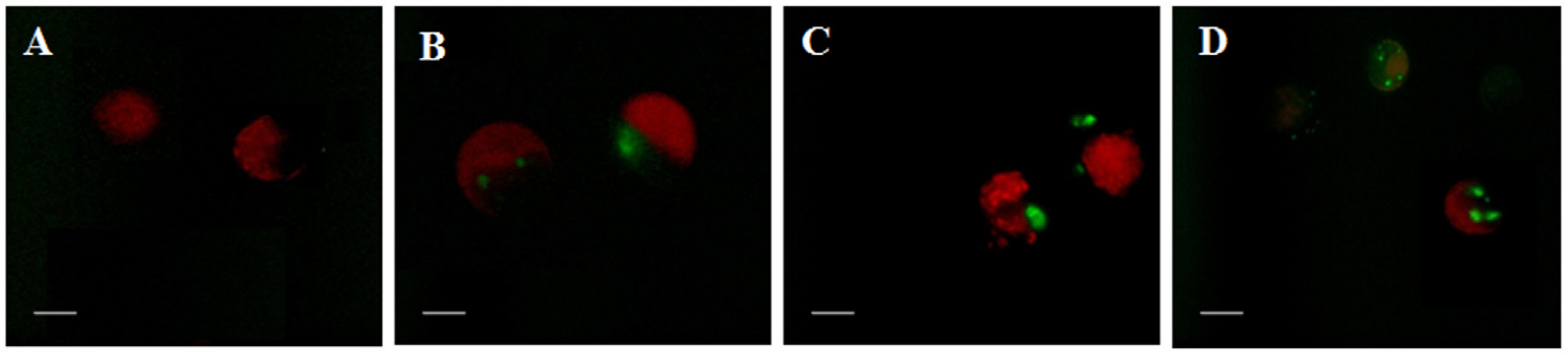
Expression of GFP in the transformed *Dunaliella* cells mediated by *Agrobacterium* strains after 8 weeks of co-cultivation. (A) Wild-type cells, (B) Transformants mediated by EHA105, (C) Transformants mediated by GV3101, (D)Transformants mediated by LBA4404. Bars equals 20 μm.

Transformants and wild-type strains were visualized under a fluorescent microscope for the detection of GFP reporter gene expression through *Agrobacterium* strains EHA105, GV3101, and LBA4404. All transformants emitted a distinctive greenish fluorescence in the cells which is characteristic of GFP. The greenish fluorescence was completely absent in wild-type cells which were treated similarly. However, autofluorescence (red color) was observed in wild-type, which may have been due to a partial evacuation of chlorophyll (Fig 4).

### Analysis of putative transgene in the genome of *Dunaliella* cells by PCR and Southern blot

**Fig 5:**
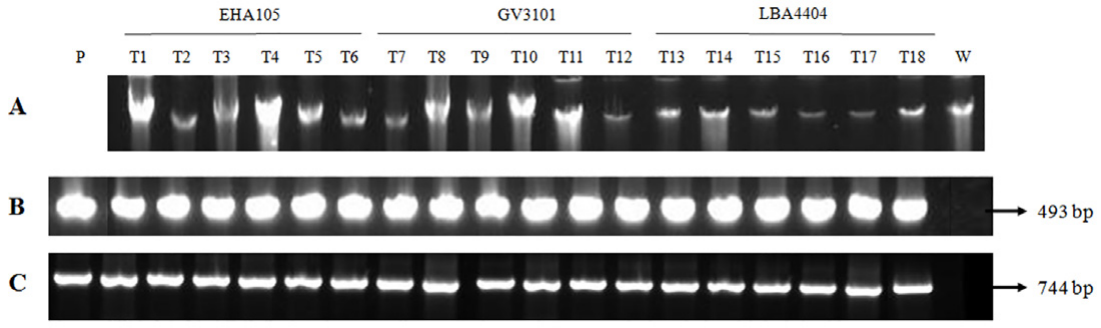
Detection of the putative transgene from genomic DNA of transformants and wild type using GFP and HPT primers. (A) Genomic DNA of transformants mediated by *Agrobacterium* strains EHA105 (T1–T6), GV3101 (T7–T12), LBA4404 (T13-T18) and Wild-type (W). The gel shows the PCR amplification of GFP fragment of size 493bp (B) and HPT fragment of size 744bp (C) in the transformants.

PCR amplification of transgene fragments of GFP (493 bp) and HPT (744 bp) using gene specific primers indicated the presence of the genes in the genomic DNA of randomly selected transformants (T1–T18) (Fig 5A) whereas no amplification was observed with DNA from wild-type cells (W). Plasmid pMDC45 (P) containing both GFP and HPT fragments was used as a positive control. Thus, the result indicates the presence of transgene GFP (Fig 5B) and HTP (Fig 5C) within the genome of transformed *Dunaliella* cells (Fig 5). The presence of the transgene in the genome of transformants was clearly indicated by PCR and the copy number was obtained using the Southern blot analysis.

**Fig 6:**
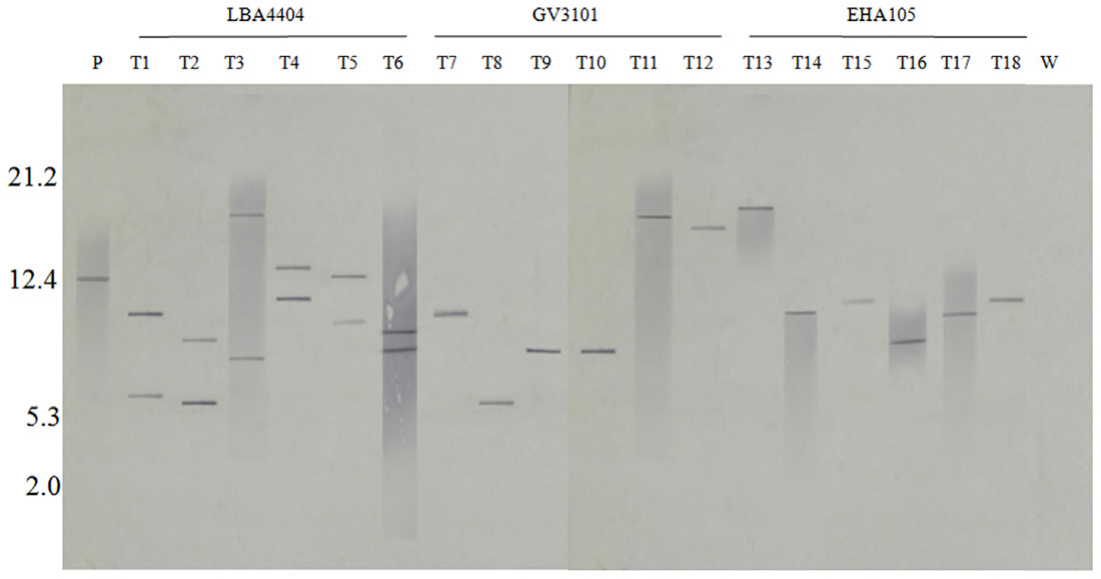
Southern blot analysis of transformants for the presence of the transgene. The transformants mediated by *Agrobacterium* strains LBA4404 (T1–T6), GV3101 (T7–T12), EHA105 (T13-T18), Wild-type (W), Plasmid pMDC45 (P). 5 μg DNA of transformants and wild-type was digested with KpnI and hybridized with a DIG-labeled HPT probe.

A Southern blot of plasmid DNA pMDC45 (P), DNA from Transgenic lines mediated by each *Agrobacterium* (LBA4404: T1–T6, GV3101: T7–T12 and EHA105: T13-T18) and wild-type cells (W) digested with *KpnI* and probed with the HPT gene fragment is depicted in Fig 6. Single *KpnI* site, which is at 2767 bp of T-DNA region and an expected a release of, is more than 5.1 kb in size as per the sequence of pMDC45 that contains the probe region of HPT. In our results, we observed two copies of the fragments in the transgenic lines mediated by LBA4404 and a single copy of the fragment in transgenic lines mediated by GV3101 and EHA105. No fragment was observed in the lane containing the DNA of the wild type cells digested with the same enzyme. The integration of the transgene copy number (HPT) in the transformed algal genome mediated by the three different *Agrobacterium* strains is given in Table 2.

**Table 2:** Integration of number of transgene (HPT) in the genome of transgenic lines mediated by each *Agrobacterium* strains as analyzed by Southern blot hybridization.

*Agrobacterium* strains	Transgenic lines with two copies	Transgenic lines with single copy	No result	Total transgenic lines
EHA105	0	15	3	18
GV3101	0	13	3	16
LBA4404	16	2	1	19

### Detection of *Agrobacterium* contamination in the transgenic lines

**Fig 7:**
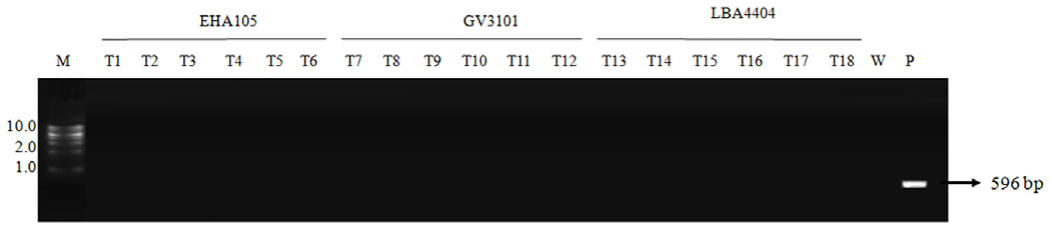
Screening of *Agrobacterium* contaminants in the transgenic lines. The transformants mediated by *Agrobacterium* strains LBA4404 (T1–T6), GV3101 (T7–T12), EHA105 (T13-T18) and Wild-type (W), Plasmid pMDC45 (P), 1 kb Marker in kb (M). The gel shows the corresponding 596 bp fragments obtained with kanamycin-specific primers.

All the transformants used for molecular characterization had been additionally assessed for the presence of bacterial contamination. Preliminary screening of the transformants involved the production of sub-cultures in enriched medium (LB) to check for the possibility of bacterial contamination as bacteria tends to grow at a faster rate than the algal cells [23]. Additional screening was carried out by performing PCR using primer specific for kanamycin which lies external to T-DNA. No amplification was noticed in the genome of the transformants (T1–T18). This data indicates the absence of *Agrobacterium* strain within the transformants (Fig 7).

### Long-term stability of DNA integration and gene expression in transgenic *Dunaliella*

Transgenic *Dunaliella* cells (0.8 to 1.0 OD at 520 nm) which were able to retain their phenotype and grow well when exposed up to 8 mg/L of hygromycin were also able to tolerate when hygromycin concentration was increased up to 16 mg/L, after a six months of sequential subculture in TAP medium (S4 Table). S2 Fig also shows the presence of HPT transgene in the genome of hygromycin resistant microalgae. Thus, the results indicated that stable integration and expression of heterologous transgene mediated by *Agrobacterium* strains without the addition of antibiotics.

## Discussion

This study describes the procedure for the development of an efficient *Dunaliella* transformation system using pre-induced *Agrobacterium* strain with D-glucose and AS. Induction of virulence in *Agrobacterium* has been well-known and demonstrated with various low molecular weight compounds such as phenolic compounds (AS) and monosaccharides (glucose) [24]. He and coworkers [17] provided a direct evidence that ChvE specifically binds to the *Vir* gene-inducing sugar D-glucose with high affinity in the presence of AS. The ChvE protein is homologous to several periplasmic sugar-binding proteins of *E*.*coli*. A further mutation in ChvE associated with the latter two phenotypes lies in two overlapping solvent-exposed site adjacent to the sugar-binding cleft. This may bring about an antagonistic impact on ChvEs interaction with its distinct protein. Currently available data indicate that under acidic conditions, plant-derived compounds are able to trigger *Vir* genes which are involved in the transfer of T-DNA [25–28]. In *Chlamydomonas*, *Agrobacterium-*mediated transformation was achieved with the help of 1 mM glycine betaine with AS at a pH of 5.2 [9]. In our study, we also used different concentrations of D-glucose (5 mM, 10 mM, and 15 mM) with AS to trigger the *Vir* genes of Agrobacterium strains for infection in the *Dunaliella* cells.

The growth of *D*. *salina* was completely suppressed at a hygromycin concentration of 3 mg/L of in TAP medium. Thus, the minimal inhibitory concentration of hygromycin for *D*. *salina* was much lower as compared to those reported in previous studies for *D*. *bardawil* (100 mg/L) [11], *Porphyra yezoensis* [29], *Parachlorella kessleri* [12] and *Schizochytrium* sp. [30]. This may be because of the facilitation of the growth of *D*. *salina* in a TAP medium with low concentration of salt (0.1M). It has been stated that a higher convergence of salt promotes the utilization of higher amount of hygromycin for the complete termination of *D*. *salina* cell development because of the inclusion of H^+^-ATPase [31]. However, the antibiotic sensitivity of many algal species has not been studied in depth and requires further investigation to pinpoint the exact mechanisms. 500 mg/L of cefotaxime was found to be inhibitory for *Agrobacterium*; however, this concentration did not affect the *D*. *salina* cells and thus was used for the elimination of the bacteria after co-cultivation. Co-cultivation studies revealed that the combined use of D-glucose and AS stimulate the virulence capacity of *Agrobacterium* strains and thus result in a significant increase in the transformation frequency of *D*. *salina* mediated by LBA4404, EHA105 and GV310 at the concentration of 10 mM D-glucose in the presence of 100 μM AS. But, D-glucose and AS alone do not cause any significant increase in the transformation rate. Several studies have reported that microalgae are able to secrete several small molecules, including phenolics into the culture medium during their growth [32–34]. It is likely that some of these molecules are capable of inducing the *Agrobacterium Vir* genes, explaining the transformation as well as *Vir* gene induction. Thus, the use of D-glucose with AS for pre-induction of *Agrobacterium* increases the transformation frequency up to 4 times that of *Agrobacterium-*mediated transformation in *Dunaliella* sp with or without AS (42 ± 3 per 10^6^ cells) [11]. Despite extensive studies on *Agrobacterium-*mediated transformation of microalgae, the data inferred on transformation frequencies between investigations vary significantly [30,35–37]. In our study, induction of the *Vir* genes in *Agrobacterium* strains by D-glucose appears to work synergistically with induction by AS.

The gene coding for the green fluorescent protein (GFP) from the *Aequorea victoria* has been utilized as a marker for gene expression [38] and in vivo protein localization in microalgae [39–41]. In this study, the expression levels of GFP in the cells also varied with the *Agrobacterium* strains. LBA4404 mediated *Dunaliella* transformants indicated a higher level of expression compared to GV3101 and EHA105 mediated transformants. Expression levels of the reporter gene may have varied due to random insertion of one or more transgenes into the genome of transgenic algae, [2]. Preliminary study revealed the presence of transgenes (HPT and GFP) in the genome of the transgenic lines. Transgene copy number may influence the levels of expression of integrated genes, gene silencing, and stability. Gene copy number is also an important parameter for plant biotechnology, since transgenic plants with single gene insertions are preferentially approved by regulatory agencies [42]. Thus, it results in a different number of transgenes in the HPT resistant transformants and this number depends on *Agrobacterium* infection on algae. Most of the transformants mediated by LBA4404 showed two copies of the transgene and those mediated by EHA105 and GV3101 showed a single copy respectively. Identical results from the prior studies reveal that single or multiple copies of transgenes can be seen from transformants mediated by EHA105, EHA101 and LBA4404 respectively in microalgae [11,30,43]. Fang and his coworkers studied the expression of sedoheptulose-1,7-bisphosphatase from *C*. *reinhardtii* in *D*. *bardawil* mediated by GV3101 but unfortunately, did not evaluate the transformation frequency of GV3101 [44]. In other algal species, LBA4404 mediated transformants contained a single-copy of T-DNA in the genome [9,35]. Differences in copy number obtained with either assay may have resulted from partial restriction digestion of genomic DNA, insertion of tandem repeats, re-arrangements or truncations of the inserted T-DNA[45–48]. A recent study indicated that the highest transformation rate was observed in GV3101 followed by EHA105, AGL1, and MP90 but the mortality rate was quite lower with the strain GV3101. Even EHA105 was more efficient in the transfer of single-copy transgene than GV3101 in the genome of tomato [42]. The outcomes presented here demonstrate that super virulent *A*. *tumefaciens* GV3101 strain seemed to have an ideal combination of transformation efficiency and capacity to create a single copy of transgene in *Dunaliella* sp. Thus, the study uncovers that *Agrobacterium* strains differ not only in their transformation proficiency and transgene copy number, but these values may vary from species to species. To our knowledge, this is the first report on pre-induction of *Agrobacterium* strains with D-glucose as an inducer in the presence of AS in *D*. *salina*.

## Supporting Information

S1 Fig After 8 weeks of co-cultivation, Individual hygromycin resistant colonies mediated by each pre-induced Agrobacterium strains (10 mM D-glucose and 100μM) in solid TAP medium containing 3 mg/L. (A) EHA105 mediated transformation, (B) GV3101mediated transformation, (C) LBA4404 mediated transformation and (D) wild type cells alone.
(DOCX)

S2 Fig Stable integration and presence of transgene (HPT) in hygromycin resistant cells mediated by pre-induced *Agrobacterium* strains. LBA4404 (T1–T6), GV3101 (T7–T12), EHA105 (T13-T18) and 1 kb Marker (M). The gel shows the corresponding 744 bp fragments obtained with hygromycin specific primers.
(DOCX)

S1 Table Description of *Agrobacterium* strains used in this study. (DOCX)

S2 Table Gene specific primers were used in this study. (DOCX)

S3 Table Effect of cefotaxime antibiotic sensitivity on *Agrobacterium* strains and *Dunaliella salina*. (DOCX)

S4 Table Growth and tolerance of wild and transgenic Dunaliella cells in the TAP medium containing different concentration of hygromycin. Hygromycin resistant phenotypes are able to grow when exposed upto 8 mg/L and also tolerant upto 16 mg/L of selective antibiotic containing medium.
(DOCX)
