# Supplementary material for: Synergistic Action of D-Glucose and Acetosyringone on Agrobacterium Strains for Efficient Dunaliella Transformation
Source: PLoS One. 2016 Jun 28;11(6):e0158322. doi: 10.1371/journal.pone.0158322 (PMC4924854; doi:10.1371/journal.pone.0158322)
Supplement: S1 Fig — (A) EHA105 mediated transformation, (B) GV3101mediated transformation, (C) LBA4404 mediated transformation and (D) wild type cells alone. (DOCX) [file pone.0158322.s001.docx]

**
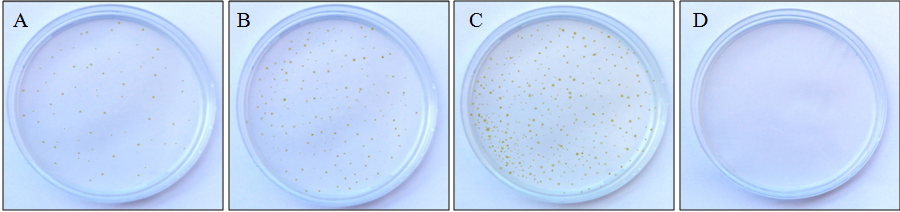
**

**S1 Fig.** **After 8 weeks of co-cultivation, Individual hygromycin resistant colonies mediated by each pre-induced Agrobacterium strains (10 mM D-glucose and 100µM) in solid TAP medium containing 3 mg/L.** (A) EHA105 mediated transformation, (B) GV3101mediated transformation, (C) LBA4404 mediated transformation and (D) wild type cells alone.
