# Supplementary material for: Synergistic Action of D-Glucose and Acetosyringone on Agrobacterium Strains for Efficient Dunaliella Transformation
Source: PLoS One. 2016 Jun 28;11(6):e0158322. doi: 10.1371/journal.pone.0158322 (PMC4924854; doi:10.1371/journal.pone.0158322)
Supplement: S2 Fig — LBA4404 (T1–T6), GV3101 (T7–T12), EHA105 (T13-T18) and 1 kb Marker (M). The gel shows the corresponding 744 bp fragments obtained with hygromycin specific primers. (DOCX) [file pone.0158322.s002.docx]

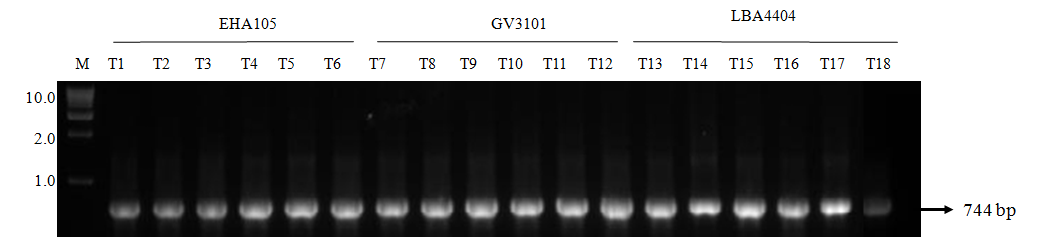


**S2 Fig.** **Stable integration and presence of transgene (HPT) in hygromycin resistant cells mediated by pre-induced *Agrobacterium* strains**. LBA4404 (T1-T6), GV3101 (T7-T12), EHA105 (T13-T18) and 1 kb Marker (M). The gel shows the corresponding 744 bp fragments obtained with hygromycin specific primers
