# Supplementary material for: Synergistic Action of D-Glucose and Acetosyringone on Agrobacterium Strains for Efficient Dunaliella Transformation
Source: PLoS One. 2016 Jun 28;11(6):e0158322. doi: 10.1371/journal.pone.0158322 (PMC4924854; doi:10.1371/journal.pone.0158322)
Supplement: S1 Table — (DOCX) [file pone.0158322.s003.docx]

**S1 Table. Description of *Agrobacterium* strains used in this study**

| **S. No.** | **Strain** | **Chromosomal background** | **Opine** | **Genome selection antibiotic** | **References** |
| --- | --- | --- | --- | --- | --- |
| 1 | GV3101 | C58 | Nopaline | Rifampicin | [1] |
| 2 | LBA4404 | TiAch5 | Octopine | Rifampicin | [2] |
| 3 | EHA105 | C58 | Succinamopine | Rifampicin | [3] |

1. Hood EE, Gelvin SB, Melchers LS, Hoekema A. New Agrobacterium helper plasmids for gene transfer to plants. Transgenic Res. 1993;2: 208–218. doi:10.1007/BF01977351

2. Hoekema A, Hirsch PR, Hooykaas PJJ, Schilperoort RA. A binary plant vector strategy based on separation of vir- and T-region of the Agrobacterium tumefaciens Ti-plasmid. Nature. 1983;303: 179–180. doi:10.1038/303179a0

3. Holsters M, Silva B, Van Vliet F, Genetello C, De Block M, Dhaese P, et al. The functional organization of the nopaline A. tumefaciens plasmid pTiC58. Plasmid. 1980;3: 212–230. doi:10.1016/0147-619X(80)90110-9
