# Supplementary material for: Synergistic Action of D-Glucose and Acetosyringone on Agrobacterium Strains for Efficient Dunaliella Transformation
Source: PLoS One. 2016 Jun 28;11(6):e0158322. doi: 10.1371/journal.pone.0158322 (PMC4924854; doi:10.1371/journal.pone.0158322)
Supplement: S2 Table — (DOCX) [file pone.0158322.s004.docx]

| Primers | Sequence (5’-3’) | Length  (bp) | Annealing  (˚C) | Amplicon  size (bp) |
| --- | --- | --- | --- | --- |
| KAN F  KAN R | GCACGAGGAAGCGGTCAGCC  GTCCGGTGCCCTGAATGAACTC | 20  22 | 60 | 569 |
| GFP F  GFP R | GCCATGTGTAATCCCAGC  GCAGTGCTTCTCCCGTTA | 18  18 | 60 | 493 |
| HPT F  HPT R | TCGGTTTCCACTATCGGCG  AGCGAGAGCCTGACCTATTGC | 19  21 | 60 | 744 |

**S2 Table. Gene specific primers were used in this study**
