# Supplementary material for: Synergistic Action of D-Glucose and Acetosyringone on Agrobacterium Strains for Efficient Dunaliella Transformation
Source: PLoS One. 2016 Jun 28;11(6):e0158322. doi: 10.1371/journal.pone.0158322 (PMC4924854; doi:10.1371/journal.pone.0158322)
Supplement: S3 Table — (DOCX) [file pone.0158322.s005.docx]

| S.No | Cefotaxime  Concentration (mg/L) | % Survival |  |
| --- | --- | --- | --- |
|  |  | Agrobacterium | Dunaliella |
| 1 | 0 | 100.00 | 100.00 |
| 2 | 100 | 36.16 | 99.10 |
| 3 | 200 | 5.68 | 99.20 |
| 4 | 300 | 0.00 | 98.20 |
| 5 | 400 | 0.00 | 98.00 |
| 6 | 500 | 0.00 | 98.00 |
| 7 | 1000 | 0.00 | 98.00 |
| 8 | 1500 | 0.00 | 98.00 |
| 9 | 2000 | 0.00 | 98.00 |

**S3 Table. Effect of cefotaxime antibiotic sensitivity on *Agrobacterium* strains and *Dunaliella salina*.**
