# Supplementary material for: Synergistic Action of D-Glucose and Acetosyringone on Agrobacterium Strains for Efficient Dunaliella Transformation
Source: PLoS One. 2016 Jun 28;11(6):e0158322. doi: 10.1371/journal.pone.0158322 (PMC4924854; doi:10.1371/journal.pone.0158322)
Supplement: S4 Table — Hygromycin resistant phenotypes are able to grow when exposed upto 8 mg/L and also tolerant upto 16 mg/L of selective antibiotic containing medium. (DOCX) [file pone.0158322.s006.docx]

| S.No | Hygromycin  Concentration (mg/L) | % Survival | |
| --- | --- | --- | --- |
|  |  | Wild type | Hygromycin resistant cells |
| 1 | 0 | 100.0 | 100.0 |
| 2 | 2 | 33.4 | 97.2 |
| 3 | 4 | 2.1 | 88.9 |
| 4 | 6 | 0.0 | 88.1 |
| 5 | 8 | 0.0 | 80.4 |
| 6 | 10 | 0.0 | 74.7 |
| 7 | 12 | 0.0 | 56.1 |
| 8 | 14 | 0.0 | 30.9 |
| 9 | 16 | 0.0 | 13.2 |
| 10 | 18 | 0.0 | 0.0 |
| 11 | 20 | 0.0 | 0.0 |

**S4 Table. Growth and tolerance of wild and transgenic Dunaliella cells in the TAP medium containing different concentration of hygromycin.** Hygromycin resistant phenotypes are able to grow when exposed upto 8 mg/L and also tolerant upto 16 mg/L of selective antibiotic containing medium.
